# Are trauma research programs in academic and non-academic centers measured by equal standards? A survey of 137 level I trauma centers in the United States

**DOI:** 10.1186/s13037-021-00309-2

**Published:** 2021-10-09

**Authors:** Robert M. Madayag, Erica Sercy, Gina M. Berg, Kaysie L. Banton, Matthew Carrick, Mark Lieser, Allen Tanner, David Bar-Or

**Affiliations:** 1 Trauma Services Department, St. Anthony Hospital, Lakewood, CO USA; 2 Trauma Research Department, Swedish Medical Center, Englewood, CO USA; 3 Trauma Services Department, Wesley Medical Center, Wichita, KS USA; 4 Trauma Services Department, Swedish Medical Center, Englewood, CO USA; 5 Trauma Services Department, Medical City Plano, Plano, TX USA; 6 Trauma Services Department, Research Medical Center, Kansas City, MO USA; 7 Trauma Services Department, Penrose Hospital, Colorado Springs, CO USA; 8 Injury Outcomes Network and Trauma Research, LLC, 501 E Hampden Ave, Englewood, CO 80113 USA

**Keywords:** Survey, Health care quality, access, and evaluation, Research

## Abstract

**Background:**

American College of Surgeons level I trauma center verification requires an active research program. This study investigated differences in the research programs of academic and non-academic trauma centers.

**Methods:**

A 28-question survey was administered to ACS-verified level I trauma centers in 11/12/2020–1/7/2021. The survey included questions on center characteristics (patient volume, staff size), peer-reviewed publications, staff and resources dedicated to research, and funding sources.

**Results:**

The survey had a 31% response rate: 137 invitations were successfully delivered via email, and 42 centers completed at least part of the survey. Responding level I trauma centers included 36 (86%) self-identified academic and 6 (14%) self-identified non-academic centers. Academic and non-academic centers reported similar annual trauma patient volume (2190 vs. 2450), number of beds (545 vs. 440), and years of ACS verification (20 vs. 14), respectively. Academic centers had more full-time trauma surgeons (median 8 vs 6 for non-academic centers) and general surgery residents (median 30 vs 7) than non-academic centers. Non-academic centers more frequently ranked trauma surgery (100% vs. 36% academic), basic science (50% vs. 6% academic), neurosurgery (50% vs. 14% academic), and nursing (33% vs. 0% academic) in the top three types of studies conducted. Academic centers were more likely to report non-profit status (86% academic, 50% non-academic) and utilized research funding from external governmental or non-profit grants more often (76% vs 17%).

**Conclusions:**

Survey results suggest that academic centers may have more physician, resident, and financial resources available to dedicate to trauma research, which may make fulfillment of ACS level I research requirements easier. Structural and institutional changes at non-academic centers, such as expansion of general surgery resident programs and increased pursuit of external grant funding, may help ensure that academic and non-academic sites are equally equipped to fulfill ACS research criteria.

**Supplementary Information:**

The online version contains supplementary material available at 10.1186/s13037-021-00309-2.

## Background

Trauma centers are categorized as level I-IV by the American College of Surgeons (ACS) according to the center’s clinical resources and leadership in research, education, and scholarly activities [1]. Level I is the highest designation, and criteria for this level include both volume thresholds (≥1200 trauma admissions per year or 240 admissions per year with Injury Severity Score > 15) and evidence of an active trauma research and scholarship program [2]. The latter criteria are evaluated primarily through publication of peer-reviewed articles generated by research studies conducted at the trauma center: during the 3-year ACS review cycle, a trauma center must publish either 20 articles in peer-reviewed journals or 10 articles in conjunction with trauma-related scholarly activities such as leadership in national organizations, resident participation in paper competitions, and support for mentorships and fellowships [2]. In addition, the trauma center must demonstrate strong structural support for the research program through availability of lab space, staff such as epidemiologists and biostatisticians, and time or financial support for clinicians to conduct research [2].

Because of the high bar set for ACS level I verification, these trauma centers serve as comprehensive regional resources that provide the highest level of specialized, immediate trauma care [3–6]. Level I status has shown to be associated with improved clinical outcomes, including lower mortality rates, lower rates of disability at discharge, and better overall outcomes in severely injured patients, compared to level II-IV centers and undesignated trauma centers [7–10]. Undergoing the process of transitioning to ACS level I status, including restructuring of the trauma department and greater oversight of patient care, has been shown to lead to improved patient outcomes, including reductions in both mortality and patient length of stay [11]. Overall, level I trauma centers serve as nationwide leaders in both patient care and clinical research.

In a 2008 survey of ACS-verified level I trauma centers, 66% reported university affiliation [4], and a 2019 study of 454 emergency departments (EDs) found that academic EDs were more likely to have ACS level I designation than non-academic EDs [12]. Academic level I trauma centers report employing more trauma surgeons and staff than level I community hospitals [4], and university-affiliated trauma centers often have a wide network of faculty, staff, and students dedicated to clinical research [13]. A 2011 study noted that because many orthopedic trauma surgeons practice outside of the academic setting, these physicians must actively seek research opportunities outside of their regular clinical duties and without support from their practice, including obtaining external funding sources [14]. Thus, it is possible that academic trauma centers are better positioned to fulfill the research requirements for ACS level I trauma center verification [6].

This study aimed to describe the trauma research programs of all ACS-verified level I trauma centers in the United States, as well as compare the abilities of academic and non-academic centers to fulfill the research requirements of the ACS. The purpose of the study was to shed light on the various methods used at level I trauma centers to establish and maintain an active research program and investigate how research goals are balanced with patient care.

## Methods

A 28-question survey (Supplementary Fig. 1) was designed with input from the Trauma Medical Directors and Clinical Research Coordinators at six ACS-verified level I trauma centers across four states. The survey was organized into five sections: 1) characteristics of the trauma center, including size and staffing; 2) subject matter and clinical specialties represented in trauma research projects; 3) research products used to fulfill ACS research requirements; 4) the effect of the COVID-19 pandemic on ongoing trauma research (results from section 4 have been published separately [15]); and 5) financial and staffing support for the trauma research program.

The primary recipients of the survey were the Trauma Program Directors or Trauma Program Managers of all ACS-verified adult level I trauma centers nationwide. If a trauma center did not have personnel in one of these positions, the survey was instead sent to the Trauma Medical Director. A list of all ACS-verified adult level I trauma centers was obtained from the ACS website; as of 11/1/2020, this list contained 175 facilities. Email contact information was obtained from individual trauma center websites and via phone contact with the facilities; only 152 of the total 175 facilities had email contact information listed online or were able to be reached via phone to obtain this information.

The survey was created and distributed through SurveyMonkey (SurveyMonkey Inc., San Mateo, CA). Survey distribution included an initial invitation sent to each center’s contact email address, with five reminder emails sent once a week to all non-respondents over the period 11/12/2020–12/17/2020, with the survey remaining open to any additional responses for 3 weeks (1/7/2021 close date). The initial invitation included an informed consent form to participate in the study and a link to opt out of the study and all subsequent survey reminders. All responses were collected anonymously, and no responses could be matched to the individual responding center. The study was determined to be non-human subjects research by the relevant Institutional Review Board.

Survey data were exported from SurveyMonkey and analyzed using SAS 9.4 (Cary, NC). Responses are reported as n (%) or median (IQR), and subgroup analyses were conducted to compare responses between academic and non-academic trauma centers using chi-square, Fisher’s exact, or Mann-Whitney U tests.

## Results

**Fig. 1:**
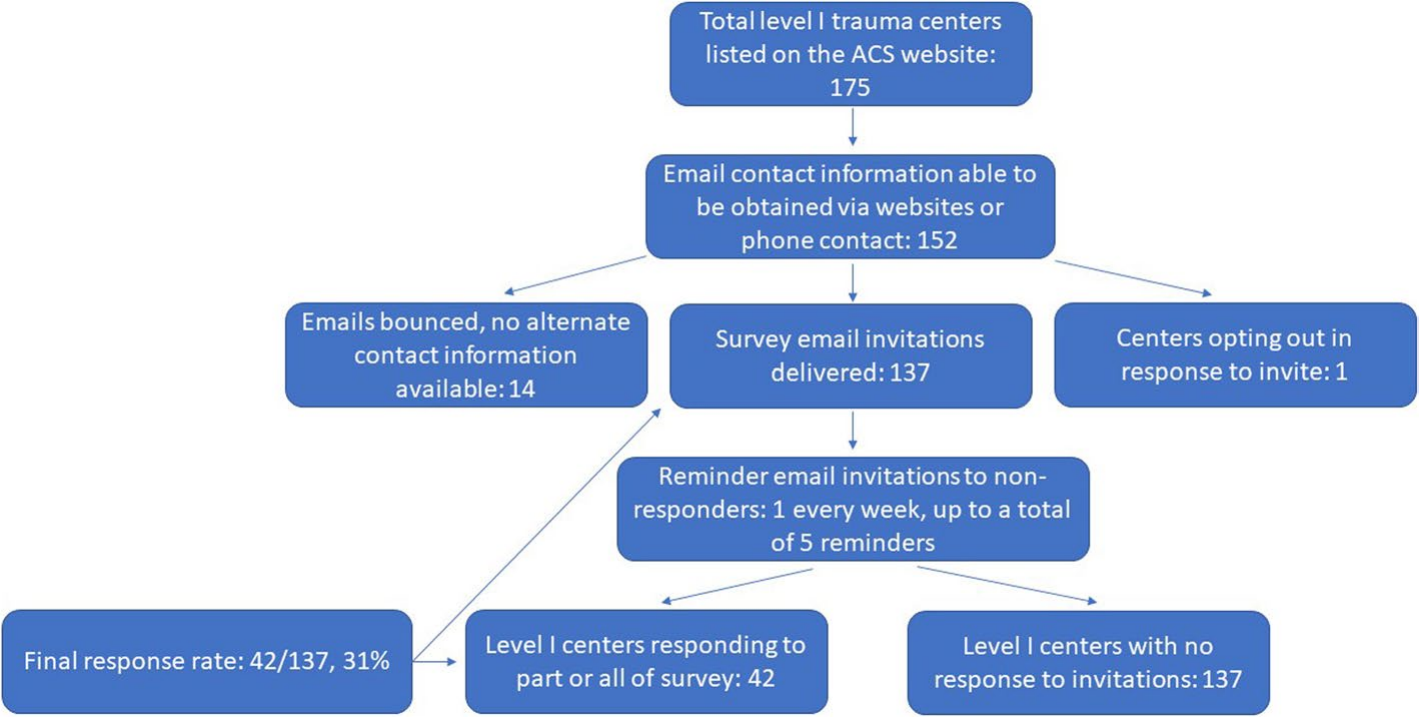
Flow diagram of study enrollment

Contact information was obtained for 152 of the 175 total level I trauma centers, and 137 survey invitations were successfully administered (14 bounced email invitations with no alternate contact information, 1 center opted out of participation). Forty-two total responses were received (27 complete, 15 partially complete), for a response rate of 31% (42/137), representing 24% of all level I trauma centers in the US (42/175) (Fig. 1).

Academic trauma centers (self-identified in the survey as an “academic or teaching hospital affiliated with a university”) comprised 86% of all respondents (*n* = 36), and non-academic centers (self-identified as a “non-academic hospital (e.g., community hospital)”) comprised 14% of respondents (*n* = 6). Academic and non-academic centers reported similar participation in a hospital system (81% academic, 100% non-academic; *P* = 0.57), numbers of licensed beds (median 545 academic, 440 non-academic; *P* = 0.21), annual trauma patient volume (median 2190 academic, 2450 non-academic; *P* = 0.95), years of ACS level I verification (median 20 academic, 14 non-academic; *P* = 0.59), concurrent state level I verification (86% academic, 100% non-academic; *P* = 1.00), and transfer patients as a percentage of the total trauma patient population (median 31% academic, 40% non-academic; *P* = 0.18) (Table 1). Academic centers were significantly more likely to be non-profit status (86% versus 50% non-academic; *P* = 0.02); of note, non-profit, government, or for-profit status was self-reported in the survey, and respondents could not choose more than one response to this question (e.g., although government or federally run hospitals are often non-profit, respondents were directed to choose the single designation that best described their center). Academic trauma centers reported significantly more full-time trauma surgeons (median 8 per year versus 6 at non-academic centers; *P* = 0.02) and general surgery residents (median 30 per year versus 7 at non-academic centers; *P* < 0.01) than non-academic centers.

**Table 1:** Characteristics of level I trauma centers

	All	Academic	Non-academic	*P*
*Profit status, total responses*	*n = 41*	*n = 35*	*n = 6*	**0.02**
Non-profit	33 (80%)	30 (86%)	3 (50%)	
For-profit	2 (5%)	0 (0%)	2 (33%)	
Government	6 (15%)	5 (14%)	1 (17%)	
*Part of a hospital system, total responses*	*n = 42*	*n = 36*	*n = 6*	0.57
n (%)	35 (83%)	29 (81%)	6 (100%)	
*Total licensed beds, total responses*	*n = 40*	*n = 34*	*n = 6*	0.21
Median (IQR), range	509 (400–710), 249–1000	545 (400–719), 249–1000	440 (330–600), 250–800	
*Approximate adult trauma admissions in the past 12 months, total responses*	*n = 40*	*n = 34*	*n = 6*	0.95
Median (IQR), range	2300 (1500–3050), 1200–4800	2190 (1460–3200), 1200–4800	2450 (1500–3000), 1500–3100	
*Full-time trauma surgeons per year (contract/employment), total responses*	*n = 41*	*n = 35*	*n = 6*	**0.02**
Median (IQR), range	8 (6–9), 3–16	8 (6–10), 3–16	6 (5–6), 3–8	
*General surgery residents per year (PGY1-PGY5), total responses*	*n = 39*	*n = 33*	*n = 6*	**< 0.01**
Median (IQR), range	30 (20–35), 0–60	30 (25–35), 10–60	7 (6–15), 0–16	
*Years of ACS level I verification, total responses*	*n = 42*	*n = 36*	*n = 6*	0.59
Median (IQR), range	20 (7–28), 1–36	20 (8–27), 1–36	14 (4–28), 3–30	
*State level I verification, total responses*	*n = 42*	*n = 36*	*n = 6*	1.00
n (%)	37 (88%)	31 (86%)	6 (100%)	
*Percentage of patients transferred in, total responses*	*n = 40*	*n = 34*	*n = 6*	0.18
Median (IQR), range	33 (20–43), 0–70	31 (20–41), 0–68	40 (35–52), 15–70	

Bolding of *P* values indicates statistical significance at a threshold of *P* ≤ 0.05

*IQR* interquartile range, *PGY* postgraduate year, *ACS* American College of Surgeons

When asked to rank 11 clinical subject areas in terms of the number of studies conducted in each area, trauma surgery (45%), orthopedic surgery (21%), critical care/critical care surgery (21%), and neurosurgery (19%) were most frequently ranked among the top three subjects studied (Table 2). Academic and non-academic trauma centers differed significantly in the subject areas that were the predominant foci of their research programs. All non-academic centers ranked trauma surgery in their top three most frequent subjects of study, whereas only 36% of academic sites did so (*P* < 0.01). Non-academic centers also had stronger focus on basic science, with 50% of non-academic sites ranking basic science in their top three research topics compared to only 6% of academic sites (*P* = 0.02), and nursing, with 33% of non-academic sites reporting nursing in their top three research topics compared to 0% of academic sites (P = 0.02).

**Table 2:** Characteristics of research programs

	All	Academic	Non-academic	*P*
*Ranked in top three study frequency, total responses*	*n = 42*	*n = 36*	*n = 6*	
Trauma surgery	19 (45%)	13 (36%)	6 (100%)	**< 0.01**
Orthopedic surgery	9 (21%)	7 (19%)	2 (33%)	0.59
Critical care/critical care surgery	9 (21%)	7 (19%)	2 (33%)	0.59
Neurosurgery	8 (19%)	5 (14%)	3 (50%)	0.07
Emergency medicine	6 (14%)	6 (17%)	0 (0%)	0.57
Basic sciences	5 (12%)	2 (6%)	3 (50%)	**0.02**
Vascular surgery	4 (10%)	4 (11%)	0 (0%)	1.00
Radiology	3 (7%)	3 (8%)	0 (0%)	1.00
Cardiothoracic surgery	2 (5%)	2 (6%)	0 (0%)	1.00
Nursing	2 (5%)	0 (0%)	2 (33%)	**0.02**
Anesthesia	0 (0%)	0 (0%)	0 (0%)	–

Bolding of *P* values indicates statistical significance at a threshold of *P* ≤ 0.05

Overall, most responding trauma centers used the traditional 20-publication route to fulfill ACS research requirements (72%) (Table 3). Among those centers that used the alternate 10-publication route, commonly reported barriers to the 20-publication route were lack of dedicated clinician research time (67%), lack of research support staff (22%), and lack of clinician interest in research (22%). When asked what specific subjects were represented among the publications used to fulfill ACS requirements from a list of 11 subjects, the most frequently reported topics were trauma surgery (100%), orthopedic surgery (82%), critical care/critical care surgery (79%), emergency medicine (75%), and neurosurgery (64%). Academic sites more frequently reported representation of orthopedic surgery (91% versus 50% of non-academic sites; *P* = 0.05) among their publications, and non-academic sites more frequently reported representation of nursing (83% versus 18% of academic sites; *P* < 0.01) among their publications.

**Table 3:** Fulfilling ACS research requirements

	All	Academic	Non-academic	*P*
*Method of fulfillment, total responses*	*n = 29*	*n = 23*	*n = 6*	1.00
Traditional 20-publications	21 (72%)	17 (74%)	4 (67%)	
Alternate 10-publications and scholarly activity	8 (28%)	6 (26%)	2 (33%)	
*Scholarly activities used in alternate route, total responses*	*n = 8*	*n = 6*	*n = 2*	
Resident participation in mentoring scholarly activity (e.g., resident paper competitions)	8 (100%)	6 (100%)	2 (100%)	–
Dissemination of knowledge (e.g., review articles, book chapters)	7 (88%)	5 (83%)	2 (100%)	1.00
Leadership in major trauma organizations	6 (75%)	4 (67%)	2 (100%)	1.00
Participation as visiting professor or invited lecturer	6 (75%)	5 (83%)	1 (50%)	0.46
Scholarly application of knowledge (e.g., case reports)	5 (63%)	4 (67%)	1 (50%)	1.00
Mentorship of fellows and maintenance of fellowships	4 (50%)	2 (33%)	2 (100%)	0.43
Peer-reviewed funding	3 (38%)	3 (50%)	0 (0%)	0.46
*Barriers to 20-publication route, total responses*	*n = 9*	*n = 6*	*n = 3*	
Lack of dedicated research time of clinicians	6 (67%)	4 (67%)	2 (67%)	1.00
Lack of support staff dedicated to research	2 (22%)	1 (17%)	1 (33%)	1.00
Lack of interest of participation of a sufficient number of clinicians	2 (22%)	1 (17%)	1 (33%)	1.00
Lack of compensation for scholarly activities or time spent on research	1 (11%)	0 (0%)	1 (33%)	0.33
*Specialties represented in publications, total responses*	*n = 28*	*n = 22*	*n = 6*	
Trauma surgery	28 (100%)	22 (100%)	6 (100%)	–
Orthopedic surgery	23 (82%)	20 (91%)	3 (50%)	**0.05**
Critical care/critical care surgery	22 (79%)	17 (77%)	5 (83%)	1.00
Emergency medicine	21 (75%)	17 (77%)	4 (67%)	0.62
Neurosurgery	18 (64%)	16 (73%)	2 (33%)	0.15
Basic sciences	12 (43%)	9 (41%)	3 (50%)	1.00
Radiology	9 (32%)	7 (32%)	2 (33%)	1.00
Nursing	9 (32%)	4 (18%)	5 (83%)	**< 0.01**
Vascular surgery	6 (21%)	6 (27%)	0 (0%)	0.29
Anesthesia	3 (11%)	3 (14%)	0 (0%)	1.00
Cardiothoracic surgery	0 (0%)	0 (0%)	0 (0%)	–

Bolding of *P* values indicates statistical significance at a threshold of *P* ≤ 0.05

All responding trauma centers reported using internal funding for trauma research, 63% additionally used external governmental or non-profit grants, and 30% also received corporate or private sponsorship (Table 4). Significantly more academic trauma centers reported receiving external grants (76% versus only 17% of non-academic centers; *P* = 0.02). The most commonly reported research-related compensation available to the Trauma Medical Director was financial compensation for research activities, such as reimbursement for conference attendance (71%). Other forms of compensation, such as dedicated time for research (41%) or financial compensation for dedicated research time (7%), were less common. A majority of trauma centers reported having clinical research or study coordinators (73%), student employees or volunteers (65%), biostatisticians (62%), and an Institutional Review Board coordinator (50%) available when performing trauma research. Less than half of sites reported an available epidemiologist (35%), basic scientists (31%), dedicated research workspace (31%), dedicated lab space (31%), and grant writers (15%). Most centers reported that the staff and workspace used for research were provided by the hospital (85%), with 33% reporting that these resources were supplied by the university affiliated with the hospital, and 22% reporting they were supplied by an external research partner. Overall, only 56% of trauma centers reported the presence of staff dedicated solely to research who had no hospital administrative tasks or clinical duties.

**Table 4:** Financial and staff support for research

	All	Academic	Non-academic	*P*
*Funding sources for research, total responses*	*n = 27*	*n = 21*	*n = 6*	
Internal	27 (100%)	21 (100%)	6 (100%)	–
External governmental or non-profit grants	17 (63%)	16 (76%)	1 (17%)	**0.02**
Corporate/private sponsorship	8 (30%)	6 (29%)	2 (33%)	1.00
*Compensation for trauma medical director, total responses*	*n = 14*	*n = 11*	*n = 3*	
Financial compensation for research activities (e.g., conference attendance)	10 (71%)	7 (63%)	3 (100%)	0.51
Dedicated time for research	6 (43%)	5 (45%)	1 (33%)	1.00
Financial compensation for dedicated research time	1 (7%)	1 (9%)	0 (0%)	1.00
*Available personnel for research, total responses*	*n = 26*	*n = 20*	*n = 6*	
Clinical research coordinators or study coordinators	19 (73%)	14 (70%)	5 (83%)	1.00
Student employees or volunteers	17 (65%)	14 (70%)	3 (50%)	0.63
Biostatisticians	16 (62%)	13 (65%)	3 (50%)	0.64
Institutional Review Board coordinator	13 (50%)	10 (50%)	3 (50%)	1.00
Epidemiologists	9 (35%)	7 (35%)	2 (33%)	1.00
Basic scientists	8 (31%)	7 (35%)	1 (17%)	0.63
Dedicated workspace for study staff and/or data abstraction	8 (31%)	6 (30%)	2 (33%)	1.00
Dedicated laboratory for basic science research	6 (23%)	5 (25%)	1 (17%)	1.00
Grant writers	4 (15%)	3 (15%)	1 (17%)	1.00
*Research staff and workspace provided by, total responses*	*n = 27*	*n = 21*	*n = 6*	
Hospital	23 (85%)	17 (81%)	6 (100%)	0.55
Hospital-affiliated university	9 (33%)	9 (43%)	0 (0%)	0.07
External research partner (e.g., independent research company)	6 (22%)	4 (19%)	2 (33%)	0.59
*Presence of dedicated research staff (*i.e.*, staff with no hospital administrative tasks or patient care), total responses*	*n = 27*	*n = 21*	*n = 6*	0.66
n (%)	15 (56%)	11 (52%)	4 (67%)	

Bolding of *P* values indicates statistical significance at a threshold of *P* ≤ 0.05

## Discussion

Although every trauma center verified as level I by the ACS must meet all elements in a comprehensive review, including requirements for structural organization, specialized trauma care, and scholarly leadership, not all trauma centers have equal ability to devote resources to research while still providing top-notch clinical and patient care. The survey results here show that academic trauma centers may be better equipped than non-academic centers to support a robust trauma research program because of greater availability of staff and financial resources.

Academic centers reported significantly more full-time trauma surgeons and general surgery residents per year than non-academic centers. The presence of residents may help drive the development of research studies and production of peer-reviewed publications. Previous studies have found that large proportions (40–90%) of general surgery residents interrupt their clinical duties to pursue research opportunities or regularly receive protected time for research [16–20], the Accreditation Council for Graduate Medical Education (AC-GME) mandates that residents of all specialties must participate in scholarly activity [21], and general surgery residents at many academic medical centers have mandatory research requirements, including initiation of research projects and presentations at national conferences [22–24]. Thus, the presence of significantly more general surgery residents suggests increased personnel and resources devoted to the pursuit of research and scholarly activities at academic centers.

Non-academic trauma centers reported more research focus on trauma surgery, basic sciences, and nursing compared to academic centers, and a significantly greater percentage of non-academic centers reported that nursing was represented in their peer-reviewed publications (83% vs 18% of academic centers). The finding regarding focus on basic science is interesting, given that a greater proportion of academic centers reported having dedicated laboratory space for basic science research (25% compared to 17% of non-academic centers). This may imply that dedicated space does not translate directly into research interest or completion of research projects at these academic centers, or perhaps that academic centers share lab space with other university departments such as biology or chemistry.

Funding resources were also significantly different between the responding academic and non-academic trauma centers. All centers responded that they used internal funding to support their research programs. However, 76% of academic centers also leveraged external governmental or non-profit grants, whereas only 17% of non-academic centers utilized this funding source. Clinicians practicing at academic trauma centers are likely also faculty members at the hospital-associated university, and as such, are frequently urged or required to secure a portion of their salaries via external research funding, including grants from funders such as the National Institutes of Health or National Science Foundation [25–27]. Clinician faculty members may also have built-in institutional support when preparing and submitting grants [25, 28, 29]. Although the pressure to secure external funding undoubtedly adds to the burden of duties clinician faculty members at academic trauma centers face, these additional financial resources also likely result in increased staff and monetary resources available to pursue trauma research projects.

Limitations to the study include potential participation bias and a lower-than-ideal response rate. Although the response rate to this survey may be considered low (31%, or 24% of all level I centers), it is comparable to previous studies and reviews examining physician survey response rates, which ranged from 10 to 35%, as well as observations that nurses and physicians generally respond to surveys at lower rates than the general population [30–33]. Although in line with previous studies, the relatively low response rate here may have resulted in responses that were non-representative of all level I trauma centers nationwide. The median bed size and participation in a hospital system were comparable here to a previous nationwide study of ACS-verified level I trauma centers, although among respondents to this study, academic and non-profit trauma centers were overrepresented compared to previous studies [4, 6]. It is possible that centers with more staff and resources devoted to trauma research had more time and were more likely to respond than centers whose clinical staff is already overburdened with research and clinical work; on the other hand, centers with limited resources available for research may also have been motivated to respond to highlight their concerns. It is difficult to predict how participation bias may have affected the study results. However, the survey received sufficient responses to detail the research programs at approximately a quarter of all ACS-verified level I trauma centers nationwide.

## Conclusions

Results from this nationwide survey of ACS-verified level I trauma centers show that academic and non-academic trauma centers are differently equipped to fulfill the ACS research requirements for level I verification. To increase focus on trauma research programs at non-academic level I trauma centers, a range of structural and institutional changes may be worth considering. General surgery programs may be expanded to include more residents per year, providing a larger engine to drive the generation of study ideas and completion of research projects. Increased focus on securing external grant funding to supplement internal support at non-academic centers will likely result in additional resources through the full process of conducting research studies; however, because preparation of grant applications would require additional time commitment on the part of clinicians, the institution should make dedicated research time and financial support of clinicians through this process a priority. Such changes that shift resources and prioritization toward research at non-academic trauma centers may help ensure that non-academic and academic trauma centers enter the ACS verification process on equal footing.

## Supplementary Information

**Additional file 1.**

## Data Availability

The datasets used and analyzed during the current study are available from the corresponding author on reasonable request.

## Abbreviations

AC-GME: Accreditation Council for Graduate Medical Education
ACS: American College of Surgeons
ED: Emergency department
IQR: Interquartile range
