# Supplementary material for: Are trauma research programs in academic and non-academic centers measured by equal standards? A survey of 137 level I trauma centers in the United States
Source: Patient Saf Surg. 2021 Oct 9;15:34. doi: 10.1186/s13037-021-00309-2 (PMC8501921; doi:10.1186/s13037-021-00309-2)
Supplement: Supplementary file 1 — Additional file 1. [file 13037_2021_309_MOESM1_ESM.docx]

*Survey content:*

**Fulfilling the research criteria for American College of Surgeons level I trauma center verification and the effects of COVID-19 on ACS verification: A nationwide survey**

Section I: Description of your level I trauma center

1. What type of facility is your hospital?
   1. Academic or teaching hospital affiliated with a university
   2. Non-academic hospital (e.g., community hospital)
2. What is the tax status of your hospital?
   1. Non-profit
   2. For-profit
   3. Government
   4. Other (please specify):
3. Is your facility part of a hospital system?
   1. Yes
   2. No
4. Approximately how many total licensed beds are in your hospital?

_______

1. In the past year, approximately how many adult (age 18+) trauma patients were admitted to your hospital?

_______

1. How many full-time trauma surgeons does your hospital host in a typical year, through contract or employment?

_______

1. How many general surgery residents does your hospital host in a typical year (i.e., PGY1-PGY5 combined)?

_______

1. How many years have you been an ACS-verified level I trauma center?

_______ years

1. Are you also verified by your state as a level I trauma center?
   1. Yes
   2. No
2. Approximately what percentage of admitted trauma patients are transferred in from another facility?

_______

Section II: Types of trauma research performed at your level I trauma center

1. Approximately how many trauma-related research projects are conducted per year at your facility in the following categories?

____ Investigator initiated

­­­____ Externally initiated (e.g., multicenter trials as part of AAST, EAST, etc.)

____ Sponsored studies (e.g., pharmaceutical funded)

1. Please rank the following specialties by volume of research studies at your facility. Please assign each specialty a rank ranging from 1 (many studies, high research focus) to 11 (few or no research studies or focus):

____ Trauma surgery

____ Basic sciences

____ Neurosurgery

____ Orthopedic surgery

____ Emergency medicine

____ Critical care/critical care surgery

____ Radiology

____ Anesthesia

____ Vascular surgery

____ Cardiothoracic surgery

____ Nursing

____ Other (please specify):

Section III: Research products used to fulfill ACS level I requirements

1. How did your facility fulfill the ACS level I research requirements during your last ACS review?
   1. Traditional 20-publication route
   2. Alternate 10-publication plus scholarly activity route
2. If you used the alternate route on your most recent ACS review, which of the following scholarly activities did you utilize (four of the following seven are required; please select all that apply)?
   1. Leadership in major trauma organizations
   2. Peer-reviewed funding
   3. Dissemination of knowledge, including review articles, book chapters, etc.
   4. Scholarly application of knowledge, e.g., case reports
   5. Participation as a visiting professor or invited lecturer at national or regional trauma conferences
   6. Resident participation in mentoring scholarly activity, e.g., resident paper competitions
   7. Mentorship of fellows and maintenance of fellowships
3. If you used the alternate route, what are some barriers you experienced to using the 20-publication route?
   1. Lack of support staff dedicated to research
   2. Lack of dedicated research time of clinicians, including attending surgeons and residents
   3. Lack of compensation for scholarly activities or time spent on research
   4. Lack of interest or participation of a sufficient number of clinicians in research
   5. Other (please specify): _______
4. In your most recent ACS review, please select the specialties that were represented in your publications (please select all that apply):
   1. Trauma surgery
   2. Basic sciences
   3. Neurosurgery
   4. Orthopedic surgery
   5. Emergency medicine
   6. Critical care/critical care surgery
   7. Radiology
   8. Anesthesia
   9. Vascular surgery
   10. Cardiothoracic surgery
   11. Nursing
   12. Other (please specify):

Section IV: Effect of COVID-19 on ACS verification and research activities

1. Have your trauma service admission numbers been reduced during the COVID-19 pandemic period?
   1. Yes
   2. No
2. If yes, have your trauma admission numbers been reduced to levels that may no longer surpass the ACS-required volume for level I verification (1,200/year total or 240/year with Injury Severity Score >15)?
   1. Yes
   2. No
3. Was your center scheduled for an ACS review during the period March-December 2020?
   1. Yes
   2. No
4. Has your center diverted resources from research to patient care during the COVID-19 pandemic period?
   1. Yes
   2. No
5. Do you have ongoing consenting studies that have been halted or postponed because of the COVID-19 pandemic?
   1. Yes
   2. No
6. Consenting studies comprised approximately what percentage of your total research studies in your most recent ACS review?
   1. 0-25%
   2. 26-50%
   3. 51-75%
   4. >75%
7. Have diversion of resources, halting of consenting studies, or other activities related to the COVID-19 pandemic made it difficult to fulfill the research requirements of the ACS during the current review period?
   1. Yes
   2. No

Section V: Institutional support for trauma-related research activities

*Section Va: Financial support*

1. What funding sources are utilized for your trauma-related research studies (please select all that apply)?
   1. Internal funding
   2. External governmental or non-profit grants
   3. Corporate or private sponsorship
   4. Other (please specify):
2. What type of compensation is the Trauma Medical Director at your facility provided with (please select all that apply)?
   1. Dedicated time for research
   2. Financial compensation for dedicated research time
   3. Financial compensation for research activities (e.g., conference attendance)

*Section Vb: Staffing support*

1. Which of the following staff are available to you when performing trauma-related research studies (please select all that apply)?
   1. Clinical research coordinators or study coordinators
   2. Epidemiologists
   3. Biostatisticians
   4. Basic scientists
   5. Dedicated laboratory for basic science research
   6. Dedicated workspace for study staff and/or data abstraction
   7. Grant writers
   8. Institutional Review Board coordinator
   9. Student employees or volunteers
2. The research staff described in the previous question are typically provided by (please select all that apply):
   1. Your hospital
   2. The hospital-affiliated university
   3. An external research partner, e.g., an independent research company
   4. Other (please specify):
3. Are there any members of the research team dedicated solely to research, meaning that they do not also perform hospital administrative tasks or patient care?
   1. Yes
   2. No
